# Macrophage Activation by Ursolic and Oleanolic Acids during Mycobacterial Infection

**DOI:** 10.3390/molecules200814348

**Published:** 2015-08-06

**Authors:** Sonia López-García, Jorge Ismael Castañeda-Sanchez, Adelina Jiménez-Arellanes, Lilia Domínguez-López, Maria Eugenia Castro-Mussot, Javier Hernández-Sanchéz, Julieta Luna-Herrera

**Affiliations:** 1 Departamento de Inmunología, Escuela Nacional de Ciencias Biológicas, IPN, Prolongación de Carpio y Plan de Ayala S/N, 11340 México City, Mexico; E-Mails: zonyalg@yahoo.com.mx (S.L.-G.); ldmguez@yahoo.com.mx (L.D.-L.); maru278@hotmail.com (M.E.C.-M.); 2 Departamento de Genética y Biología Molecular, CINVESTAV, Avenida Instituto Politécnico Nacional Número 2508, 07360 México City, Mexico; E-Mail: jahesa7591@gmail.com; 3 Departamento de Sistemas Biológicos, Universidad Autónoma Metropolitana, Unidad Xochimilco, Calzada del Hueso Número 1100, 04960 México City, Mexico; E-Mail: jorge.encb@yahoo.com.mx; 4 Centro Médico Nacional Siglo XXI, IMSS, Unidad de Investigación Médica en Farmacología, Avenida Cuauhtémoc Número 330, 06725 México City, Mexico; E-Mail: adelinajim08@prodigy.net.mx

**Keywords:** tuberculosis, oleanolic acid, ursolic acid, triterpenes, macrophages, nitric oxide (NO), reactive oxygen species (ROS), tumor necrosis factor (TNF-α), transforming growth factor (TGF-β), CD36, TGR5

## Abstract

Oleanolic (OA) and ursolic acids (UA) are triterpenes that are abundant in vegetables, fruits and medicinal plants. They have been described as active moieties in medicinal plants used for the treatment of tuberculosis. In this study, we analyzed the effects of these triterpenes on macrophages infected *in vitro* with *Mycobacterium tuberculosis* (MTB). We evaluated production of nitric oxide (NO), reactive oxygen species (ROS), and cytokines (TNF-α and TGF-β) as well as expression of cell membrane receptors (TGR5 and CD36) in MTB-infected macrophages following treatment with OA and UA. Triterpenes caused reduced MTB growth in macrophages, stimulated production of NO and ROS in the early phase, stimulated TNF-α, suppressed TGF-β and caused over-expression of CD36and TGR5 receptors. Thus, our data suggest immunomodulatory properties of OA and UA on MTB infected macrophages. In conclusion, antimycobacterial effects induced by these triterpenes may be attributable to the conversion of macrophages from stage M2 (alternatively activated) to M1 (classically activated).

## 1. Introduction

**Figure 1:**
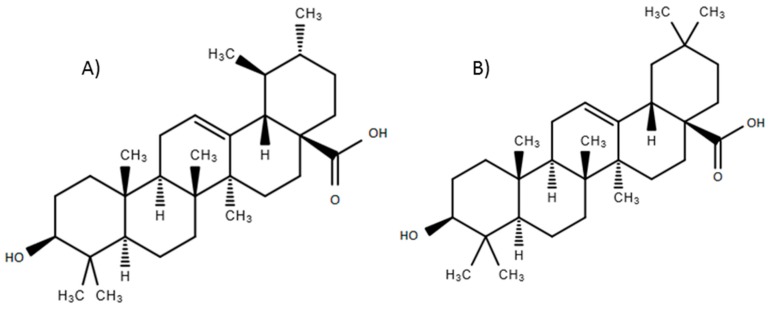
Chemical structure of ursolic (**A**) and oleanolic (**B**) acids.

Oleanolic acid (OA) ((3β-hydroxyolean-12-en-28-oic acid) and its isomer ursolic acid (UA) (3β-hydroxyurs-12-en-28-oic acid) (Figure 1), are triterpenes frequently found in vegetables and medicinal plants, as well as in several fruits such as *Chaenomeles* fruit [1] apple peel [2] and olives, among others [3]. These triterpenes are part of the human diet and medicinal use of several plants. Several studies have reported their diverse biological activities [4], including the antimycobacterial activity of several plants from different geographical localities containing these compounds [5,6,7]. Additional biological activities have been reported for these molecules, including antibacterial [8,9], antiviral [10], antiparasitic [9], antioxidant, antitumor, anti-inflammatory [11], and hepatoprotective effects [12]. UA and OA have been isolated from diverse plants, such as *Chamaedora tepejilote* [13], *Lantana hispida* [5], *Ledum groenlandicum* [11], and *Uncaria rhynchophylla* [14], among others.

TB is rampant, particularly in the developing countries. In 2013, nine million people suffered active tuberculosis (TB), including 1.1 million cases among people infected with HIV; 1.5 million people also died from TB. In this year, there was also an estimated 480,000 cases of multidrug-resistant TB (MDR-TB) among notified TB cases in patients with pulmonary TB, and an estimated of 280,000 deaths from MDR-TB [15]. Drug resistance in MTB has prompted extensive research for the discovery of antituberculosis drugs of natural origin [16]. Out of many initial leads showing *in vitro* activity, only a few of these compounds have been tested in macrophages or *in vivo* models of pre-clinical studies [7,16]. The *in vitro* minimal inhibitory concentration (a function of antibacterial activity) of plant-derived natural products UA and OA against mycobacteria has been shown to be in the range of 12.5–100 and 25–50 µg/mL, respectively [5,6,13]. These compounds are active against TB in animal models, and recently our group studied the activity of UA and OA in the BALB/c mouse TB-model infected with a drug-susceptible MTB (strain H37Rv) and MDR-MTB and observed a significant reduction of bacterial load in both types of infections [17]. In order to further understand the anti-mycobacterial characteristics of UA and OA, we evaluated their activity in a TB-macrophage model. Because the *in vitro* MIC of UA and OA were toxic to macrophages, we investigated their effect on macrophages at sub-inhibitory concentrations. At these low concentrations, we found that triterpenes exhibited immunomodulatory properties that permitted efficient control of mycobacterial growth.

## 2. Results and Discussion

### 2.1. Cytotoxicity and Intracellular Survival of Mycobacteria in J774 A.1 Cells Treated with OA and UA

**Figure 2:**
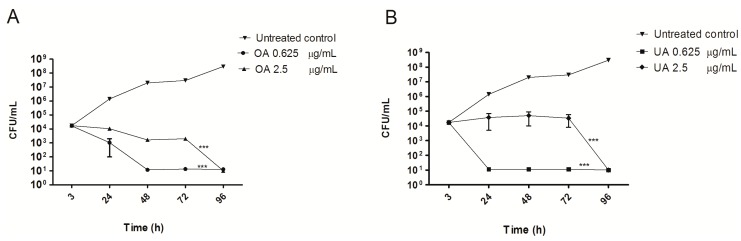
Enhancement of intracellular killing activity of macrophages against MTB by triterpenes. J774 A.1 mouse macrophages were infected with *M. tuberculosis* H37Rv for 2 h (Untreated control) and then treated with different concentrations of OA (**A**) or UA (**B**). Intracellular growth was followed up to 96 h post-infection. The results are expressed as mean ± SD from three independent experiments (*** *p* < 0.001).

To determine the optimal concentrations of OA and UA for the intracellular activity experiments, we first determined the cytotoxicity of these compounds in J774 A.1 cells. We started with *in vitro* MIC reported for both acids [5,6,17,18]. We found that these concentrations (25–50 µg/mL) were highly toxic to cells; at 24 h of treatment toxicity was above 30% and by 72 h there were almost no viable cells (data not shown). Therefore, we used sub-inhibitory concentrations ranging from 0.625–12.5 μg/mL. The cell viability was above 90% after 72 h of treatment with ≤2.5 μg/mL. Based on these observations, we used sub-toxic 0.625 and 2.5 μg/mL concentrations for macrophage treatment. Reduced cell-toxicity observed at low UA concentrations was demonstrated in *in vitro* TB-infection THP-1 cell model [19]. Both OA and UA at 2.5 and 0.625 µg/mL concentrations caused significant reduction of intracellular growth of MTB in J774 A.1 macrophages compared to untreated controls at each time point of observation (Figure 2). Both the triterpenes caused decline of intracellular colony forming units (CFU) from 10^4^ to 10^1^/mL (a 3 log reduction). On the other hand, in drug-free controls there was an increase of intracellular CFU from 10^4^ to 10^8^/mL (4 log CFU increase) by 96 h. Interestingly, lower concentrations (0.625 µg/mL) of both compounds were more effective in arresting intracellular mycobacterial growth.

### 2.2. ROS Production during Mycobacterial Infection and Triterpene Treatment

**Figure 3:**
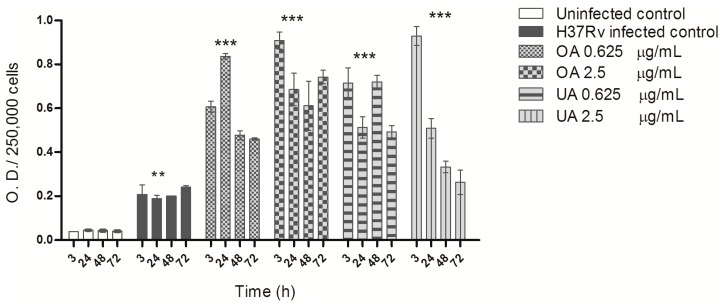
Enhancement of ROS production by triterpenes in MTB infected macrophages. ROS production was determined by the NBT reduction test. The values represent the mean ± standard deviation of three independent experiments. Statistically significant differences are indicated when infected and treated cells (UA, OA) were compared with infected cells only (H37Rv infected control) (*** *p* < 0.001) or when infected cells were compared with non-infected cells (Uninfected control) (** *p* < 0.01).

Uninfected macrophages produced low amounts of ROS; after mycobacterial infection increased ROS production slightly but significantly (Figure 3). In comparison, triterpene treatment triggered significantly higher levels of ROS. OA at both concentrations induced higher and sustained ROS production kinetics. UA at 2.5 μg/mL induced the highest ROS levels, but they decreased 24 h post-treatment, while at 0.625 μg/mL UA induced lower but sustained ROS production (Figure 3). It is known that macrophages infected with pathogenic mycobacteria produce low ROS concentrations [20,21], a finding similar to what we observed in our *in vitro* system, but when infected macrophages were treated with the triterpenes, a significant increase in ROS production was observed. Among the different biological activities described for these compounds, the anti-cancer activity demonstrated by UA and OA at high concentrations has been attributed to apoptosis due to overproduction of ROS [22,23]. In our study, low doses of UA and OA enhanced ROS production in MTB-infected macrophages, thus resulting in increased antimycobacterial activity and suppression of intracellular mycobacterial growth; a similar observation was recently reported by Podder *et al.*, with UA treatment [19].

### 2.3. NO Production during Mycobacterial Infection and Triterpene Treatment

**Figure 4:**
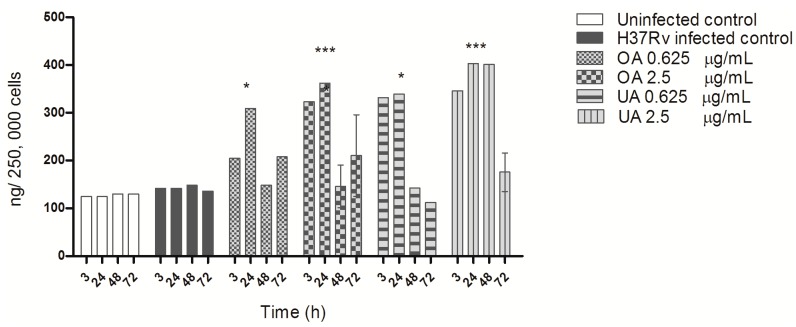
Nitric oxide production by J774 A.1 cells infected with *M. tuberculosis* and treated with triterpenes. J774 A.1 macrophages were infected with H37Rv and treated with different concentrations of triterpenes OA and UA. Nitric oxide production was determined by nitrite quantification with Griess reagent at 3, 24, 48 and 72 h post-infection. Values represent means and standard deviations from three experiments. Statistically significant differences (*** *p* < 0.001 and * *p* < 0.5) shown are, when infected and treated cells (OA, UA) were compared with infected cells (H37Rv infected control). No statistically significant differences were observed when non-infected cells (Uninfected control) were compared with infected cells (H37Rv).

Uninfected macrophages produced low NO levels, and infection with MTB did not increase NO production by J774 A.1 cells (Figure 4). In contrast, triterpene treatment induced high NO levels. Both the compounds, at both the concentration tested induced NO production in MTB-infected macrophages. However, both compounds induced higher levels of NO at 2.5 µg/mL concentrations. Peak levels of NO were detected at 24 h at 0.625 µg/mL and 3 and 24 h at 0.25 µg/mL of OA. Whereas, peak NO levels with UA were seen at 3 and 24 h at 0.625 µg/mL and up to 48 h at 2.5 µg/mL, indicating that UA is more potent in stimulating NO (Figure 4). Macrophages infected with pathogenic mycobacteria produce low amounts of NO due to the low transcription of the enzyme responsible for its production (inducible nitric oxide synthase, iNOS) [24]. Recently, UA has been shown to induce NO overproduction in a macrophage TB model [19], and earlier in an animal model of TB infection both UA and OA were shown to induce iNOS expression [17]. Our results confirm these previous observations and show that triterpene treatment induces high and early NO levels, which may be responsible or contribute to inhibition of intracellular MTB growth in macrophages [25]. Stimulation of high levels of NO by triterpenes perhaps also contribute to the anti-cancer effect of these compounds [11,26].

### 2.4. Cytokine Production during Mycobacterial Infection and Triterpene Treatment

**Figure 5:**
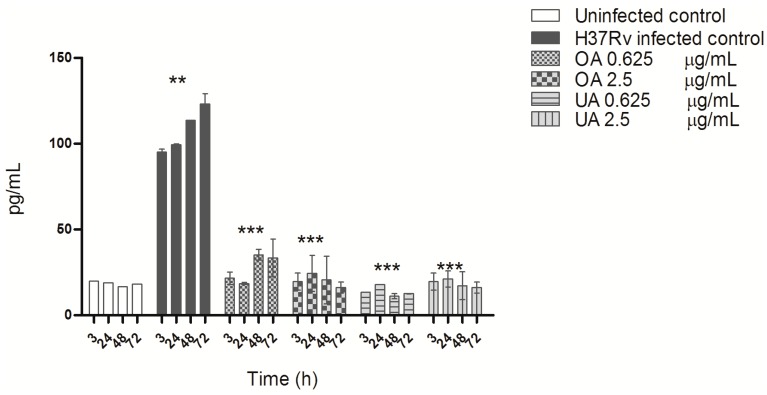
TGF-β production by macrophages infected with *M. tuberculosis* H37Rv and treated with OA and UA. Macrophages infected with strain H37Rv were treated with different concentrations of OA and UA for 3, 24, 28 and 72 h post-infection. TGF-β was quantified in cell supernatants from uninfected, infected and treated macrophages using a commercial ELISA kit. The data are presented as means ± SD from three different experiments. Statistically significant differences indicated are when infected and treated cell values (OA, UA), were compared with infected cells (H37Rv infected control) (*** *p* < 0.001), or when non-infected cells (Uninfected control) where compared with infected cells (H37Rv infected control) (** *p* < 0.001).

The effect of triterpenes, on the production of the cytokines TGF-β and TNF-α during MTB infection of macrophages was notable. Uninfected macrophages produced low amounts of TGF-β, and very high overproduction was observed 3 h post-infection and remained higher during kinetic measurements (Figure 5). In comparison, triterpene treatment induced a significant suppression of TGF-β production in MTB-infected macrophages, bringing levels back to those observed in uninfected macrophages; both concentrations of UA and OA exhibited this trend of suppression of TGF-β (Figure 5).

**Figure 6:**
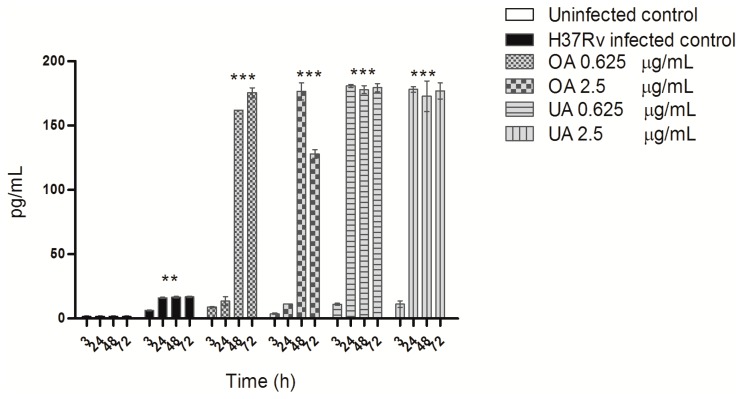
TNF-α production by macrophages infected with *M. tuberculosis* H37Rv and treated with OA and UA. Macrophages infected with strain H37Rv and treated with different concentrations of OA and UA for 3, 24, 48 and 72 h post-infection. TNF-α levels in cell supernatants from uninfected, infected and treated macrophages were quantified with a commercial ELISA kit. The data are presented as means ± SD from three different experiments. Statistically significant differences indicated are when infected and treated cells values (OA, UA), were compared with infected cells (H37Rv infected control) (*** *p* < 0.001), or when non-infected cells (Uninfected control) where compared with infected cells (H37Rv infected control) (** *p* < 0.01).

ELISA quantification of TNF-α demonstrated that untreated macrophages produced almost undetectable levels of TNF-α, and during infection with MTB a small but significant levels of TNF-α was observed (Figure 6). Oleanolic acid treatment, at both concentrations, induced high levels of TNF-α at 48 h of treatment; with ursolic acid, high levels of TNF-α were produced at 24 h of treatment (Figure 6).

Efficient control of intracellular MTB growth is mediated by activation of macrophages and Th-1 cells producing cytokines such as TNF-α and IFN-γ, both of which induce iNOS expression by macrophages and NO production [27]. When mycobacteria survive and multiply in macrophages, high levels of immunosuppressive cytokines such as TGF-β are produced [24,28]. Our study also shows a very high TGF-β (Figure 5) and a low TNF-α production in MTB-infected macrophages (Figure 6). Triterpene treatment modulated cytokine production, enhancing TNF-α production and suppressing TGF-β expression to levels similar to uninfected cells. The immunomodulatory activity of UA and OA has been described earlier, and depending on the concentration or type of experimental system, these acids can be pro-inflammatory or anti-inflammatory. Ikeda *et al.*, presented an excellent review of this issue [29]. These triterpenes are considered pro-inflammatory because they stimulate IFN-γ production [14,17] as well as upregulate iNOS and TNF-α expression through NF-κ B activation [29,30,31]. However, their anti-inflammatory activity has been described as part of suppression of TNF-α production [32] or expression of anti-inflammatory cytokines such as TGF-β [29]. In the absence of triterpene treatment, MTB-infected macrophages exhibit an M2-type of activity, whose characteristic features are low production of TNF-α, high production of TGF-β and low ROS and NO production, among others, and an inability to control mycobacterial intracellular growth [33]. Triterpene treatment reversed this phenotype to an M1-type of macrophage activity with high TNF-α production, low TGF-β production and high ROS and NO levels; activating macrophages to suppress and kill intracellular MTB [28]. 

### 2.5. Determination of Expression of CD36 and TGR5 Receptors in Macrophages Infected by M. tuberculosis H37Rv and Treated with UA and OA

**Figure 7:**
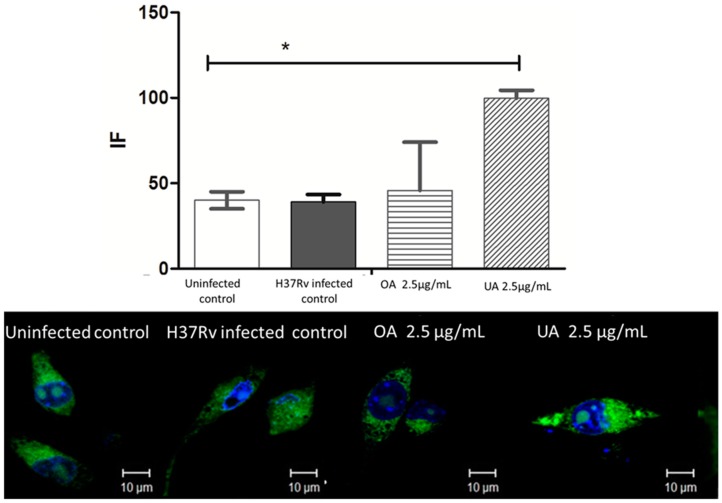
CD 36 expression on macrophages infected with MTB H37Rv and treated with OA and UA. Macrophages were infected for 2 h with MTB and then treated with 2.5 µg/mL of each triterpene. Cells were stained with anti CD36, and nuclei were stained with DAPI. Upper panel: Mean fluorescence intensity (IF) was determined in LSM Pascal V 5.1 software (Zeiss, Heidelberg, Germany). Lower panel: Representative confocal images of control macrophages (Uninfected control), infected macrophages (H37Rv infected control), infected macrophages treated with OA (OA 2.5 µg/mL) and infected macrophages treated with UA (UA 2.5 µg/mL). Green label corresponds to CD36 receptor and blue label to nuclei. Statistically significant differences are indicated (* *p* < 0.01).

It has been shown that UA and OA are recognized preferentially by two types of receptors, CD36 and TGR5, respectively [34,35]. The CD36 receptor is a class B scavenger receptor expressed on macrophages and other cell types such as endothelium and microglia [36]. This receptor binds different ligands like long-chain fatty acids, amyloid β, oxidized lipoproteins, and bacterial cell wall components, among others [37]. The precise role of CD36 in mycobacterial infections is still in study. During leprosy infection, CD36 expression is increased [38], pointing towards its role on cholesterol uptake by the macrophage and its contribution to foam cell formation. Hawkes *et al.*, in 2010, demonstrated that CD36 deficiency attenuates experimental mycobacterial infection [39]. Our results showed that: (1) J774 A.1 macrophages expressed CD36 in basal conditions; (2) after mycobacterial infection CD36 expression was increased discretely but not significantly; (3) UA treatment induced CD36 overexpression, whereas OA did not induce a significant increase of CD36 expression (Figure 7). In macrophages and dendritic cells, CD36 has been found to be associated with the innate response acting as an accessory receptor to TLR receptors including TLR2/1 and TLR2/6 heterodimers [40,41], responding to components of Gram-positive bacteria, or lipoproteins from mycobacteria. CD36 is also involved in sterile inflammation through engagement of TLR4/6 heterodimer [25]. TLR2/1, TLR2/6 or TLR4/6 heterodimer activation promoted by CD36, leads to NFκB activation and secretion of pro-inflammatory cytokines such as TNF-α, IL-6 and IL-1β, *etc.*, [25,37]. In this context, CD36 overexpression and its activation with UA treatment could be responsible for, or contribute to, the establishment of the macrophage antimycobacterial response observed. Perhaps, triterpenes acting as ligands for CD36 could synergize the TLR response, leading to the pro-inflammatory environment observed after UA treatment [34]. The engagement of any of these TLRs during triterpene treatment is an issue that should be investigated.

**Figure 8:**
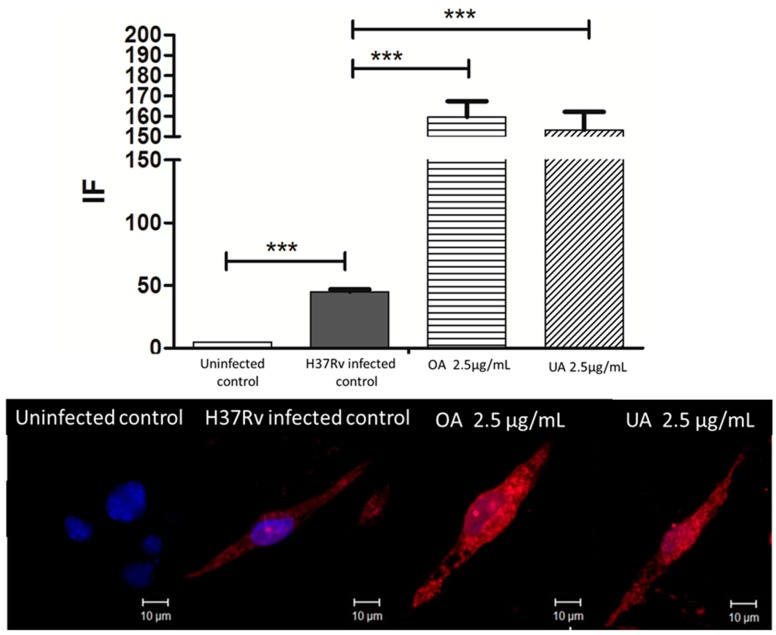
TGR5 expression in macrophages infected with MTB H37Rv and treated with OA and UA. Macrophages were infected for 2 h and then treated with UA and OA 2.5 µg/mL each for 24 h. At this time cells were stained by indirect immunofluorescence with anti-TGR5 antibody labeled with rhodamine. Upper panel) Mean fluorescence intensity was recorded with LSM Pascal V 5.1 software. Lower panel) Confocal images of control macrophages (Uninfected control); of macrophages infected with MTB H37Rv (H37Rv infected control); and macrophages infected with MTB H37Rv and treated with OA (OA 2.5 µg/mL) and UA (UA 2.5 µg/mL), respectively. The red label depicts TGR5 receptor. The blue label corresponds to cell nuclei stained with DAPI. Statistically significant differences are indicated (*** *p* < 0.001), when infected/treated cells values (OA, UA) were compared with infected cells (H37Rv), or when non-infected cells (J774 A.1) were compared with infected cells (H37Rv).

Takeda G-protein-coupled receptor 5 (TGR5) is a plasma membrane receptor coupled to G protein. Its agonists are bile acids [42,43], but also triterpenes such as betulinic, oleanolic and, to a lesser degree ursolic acid [35].TGR5 is expressed in different cells and tissues including Kupffer cells, endothelial cells, macrophages, dendritic cells, *etc.*; consequently, its activation has been associated to a great and diverse number of processes, such as those involved in metabolism, inflammation and neurodegeneration [43,44]. Some relevant processes related to metabolism, where TGR5 activation has been described to have some participation, are: energy production, oxygen consumption increase, obesity prevention, and decreasing insulin resistance [43]. TGR5-activation has been described as a potential key factor for control of diseases with inflammation as a common denominator: atherosclerosis, colitis, hepatic disease, and the metabolic syndrome [45]. It has been pointed out as a potential pharmacological target of a new generation of anti-inflammatory compounds. However, TGR-5 activation also presents potential pro-inflammatory activity through cytokine production as in IL-1β and TNF-α [46,47]. We decided to study TGR5 expression during mycobacterial infection and OA/UA treatment. We found that in basal conditions, J774A.1 macrophages expressed very low amounts of the TGR5 receptor, but mycobacterial infection triggered TGR5 cell expression (Figure 8); however, during triterpene treatment, a much higher TGR5 expression was observed (with both acids). So far there are no reports on TGR5 expression during MTB infection, nor are there reports on TGR5 expression during treatment of TB. Triterpene treatment not only changed TGR5 expression, as we showed earlier, but also modified cytokines, NO and ROS production, and triggered macrophage antibacterial capabilities. TGR5 activation by the triterpenes could be in part responsible for these features, as has been described for other macrophage models, where TGR5 activation by bile acids and triterpenes modulated their response [48,49,50]. The participation and exact role of the TGR5 receptor in tuberculosis and the macrophage response to triterpene treatment should be studied in detail.

## 3. Experimental Section

### 3.1. Reagents

UA and OA were purchased from Sigma Aldrich (St. Louis, MO, USA). Stock solutions were prepared in dimethyl sulfoxide at 1 mg/mL, and kept at −70 °C until used.

### 3.2. Cell Culture

The mouse macrophage cell model of *M. tuberculosis* infection was used [51,52,53]. Mouse macrophage J774 A.1 cell line were purchased from the American Type Culture Collection (ATCC, TIB-67, Manassas, VA, USA), grown in Dulbecco’s modified Eagle’s medium (DMEM) supplemented with 10% decomplemented fetal bovine serum, 100 units/mL penicillin and 100 µg/mL gentamicin and incubated at 37 °C in a humidified atmosphere containing 5% CO_2_. Twenty-four-well tissue culture plates were seeded with 250,000 macrophages per well in DMEM with 1% fetal bovine serum. For receptor analysis, a coverslip was set at the bottom in each well of 24-well plates.

### 3.3. Cytotoxicity and Intracellular Activity Assay

Cytotoxicity of the triterpene acids was evaluated by the trypan blue exclusion assay. Briefly, cells were treated with several concentrations of the pure compounds from 0.625 to 25 μg/mL. These preparations were made with DMEM and 1% fetal bovine serum (FBS) without antibiotics. Before treatment, wells were washed three times with warm Hanks balanced salt solution (HBSS) and triterpene solutions were added to each well. Percentage of viable cells was determined before and after 3, 24, 48 and 72 h treatment by adding trypan blue solution to reach a final concentration of 0.2%; at least 200 cells per well were counted.

### 3.4. Intracellular Antimycobacterial Activity of OA and UA

Macrophage monolayers were infected with *M. tuberculosis* strain H37Rv. Briefly, *M. tuberculosis* H37Rv was grown in Middlebrook 7H9 broth with 10% oleic acid-dextrose-catalase (OADC enrichment) until reached log phase growth. At this time, bacillary mass was washed 2 times with HBSS, adjusted to McFarland standard No. 1, and then diluted in DMEM with 1% FBS to reach a bacterial macrophage multiplicity of infection 10:1. Macrophages were incubated with the bacilli for 2 h and non-phagocyted organisms were removed by three washes with warm HBSS. Then, one mL of non-toxic concentrations of UA, OA (2.5 and 0.625 μg/mL) was added to the infected macrophages and incubated at 37 °C in a 5% CO_2_ atmosphere. At 3, 24, 48 and 72 h of treatment, the cells from each well were lysed with 0.5 mL of 0.25% sodium dodecyl sulfate solution (SDS) for 3 min and later 0.5 mL of 5% bovine serum albumin (BSA) solution was added. Control cells were incubated with DMEM without triterpenes. The viable number of bacteria (colony forming units -CFU) was determined by quantification of by plating macrophage lysates in Middlebrook 7H11 agar (Sigma Aldrich) with 10% OADC.

### 3.5. Reactive Oxygen Species (ROS) Determination

ROS were quantified according to the procedure described by Choi *et al.*, and Garcia-Perez *et al.*, [30,54]. Macrophage monolayers were prepared in ninety-six-well plates and infected for 2 h as described above. After this time, cells were treated for 3, 24, 48 and 72 h with triterpene solutions (2.5 and 0.65 μg/mL). After each time point, cells were washed three times with HBSS and then incubated with 20 μL of p-nitro-blue tetrazolium chloride solution (Sigma Aldrich, 0.1% in HBSS) at 37 °C for 20 min. Then, monolayers were washed twice with HBSS, and the formazan precipitates were dissolved by the addition of 100 μL of a solution mix of 2 M KOH and dimethyl sulfoxide (DMSO). Absorbance of soluble formazan was determined at 600 nm in a microplate reader (Labsystems, San Diego, CA, USA).

### 3.6. NO Production

Confluent J774 A.1 monolayers prepared in twenty-four-well plates were infected for 2 h as described above. After elimination of non-phagocytosed bacteria, triterpene solutions were added to the wells in 1 mL volumes at the concentrations of AU and AO of 2.5 and 0.625 μg/mL. NO production was determined at 3, 24, 48 and 72 h post-treatment. After each time point, 250 μL of cell culture medium from the corresponding wells were recovered and incubated with 50 μL of 0.8% sulfanilamide and 50 μL of 0.05% *N*-(1-naftil) ethylenediamine (Griess reactive) [30]. Nitrite production was determined at 554 nm in a microplate reader (Labsystems) and the amount of nitrite was calculated from a NaNO_2_ standard curve in HBSS established along with the experiments.

### 3.7. Quantification of Cytokines (TNF-α and TGF-β) in Supernatants by ELISA

TNF-α and TGF-β were determined in the supernatants of macrophages infected with *M. tuberculosis* H37Rv and treated with different concentrations of OA and UA with an ELISA kit according to the manufacturer’s instructions (eBioscience, San Diego, CA, USA).

### 3.8. Evaluation of CD36 and Receptor TGR5 Expression in Macrophages Infected with M. tuberculosis H37Rvand Treated with OA and UA for 24 h

J774 A. 1 cell monolayers were prepared on sterile glass coverslips by adding 150,000 cells per preparation. Cells were infected for 2 h with a mycobacteria adjusted suspension at a MOI of 10:1,then cells were washed 2 times with HBSS, followed by the addition of the triterpene solution (2.5 µg/mL). After 24 h of treatment, expression of CD36 and TGR5 receptors was analyzed by indirect immunofluorescence. Briefly, the monolayers were fixed with 4% paraformaldehyde for 30 min, washed with HBSS and incubated with 3% BSA for 30 min. After this time, they were washed three times with HBSS and a 1:350 dilution of the primary antibodies anti-CD36 (GeneTex, Irvin, CA, USA) and anti-TGR5 (Santa Cruz Biotechnology, Dallas, TX, USA), was added and incubated overnight at 4 °C. Then the monolayers were washed three times with HBSS, incubated with 3% BSA for 15 min, washed again and the secondary antibody were added at a 1:350 dilution (rabbit anti-mouse fluorescein isothiocyanate labeled (FITC) (Santa Cruz Biotechnology) and mouse anti-goat rhodamine labeled antibodies (Santa Cruz Biothecnololgy) and incubated for 2 h; finally, the monolayers were washed five times with HBSS and mounted on glass-slides using Vectashield mounting medium with 4′,6-diamidino-2-phenylindole (DAPI) counterstain (Vector Laboratories, Burlingame, CA, USA). Preparations were observed in a confocal laser scanning system attached to a microscope (LSM5 Pascal, Zeiss, Heidelberg, Germany). The results were expressed as fluorescence intensity (FI) of 200 cells analyzed in LSM5 Pascal software.

### 3.9. Statistical Analysis

All the determinations were performed in triplicate. Results were expressed as means ± standard deviations (SD) and data were analyzed using the two-way ANOVA test (Prism 5 software, GraphPad, La Jolla, CA, USA) to determine statistical significance.

## 4. Conclusions

Our results demonstrate that triterpenes the UA and OA, the two structural isomers most abundant in plants, are potent immunomodulators of macrophages infected with virulent *M. tuberculosis.* Their immunomodulatory activity induced through activation of macrophages. MTB infection of macrophages exhibited an M2-like phenotype characterized by low expression of TNF-α, high expression of TGF-β, and low production of ROS and NO as well as the inability to control mycobacterial growth. After treatment with UA and OA, the infected macrophages exhibited an M1-like phenotype characterized by sustained high production of TNF-α, consistent low production of TGF-β, high production of ROS and NO, importantly, infected macrophages were able to control and eliminate intracellular mycobacteria. The expression of CD36 and TGR5 receptors by macrophages after infection (TGR5), or after treatment (CD36 and TGR5), may explain how these triterpenes act as immunomodulators, since it is known that activation of these receptors by UA and OA in macrophages, potentiates their antimicrobial and pro-inflammatory activities. The precise signaling triggered by these triterpenes in the infected macrophages should be studied.
